# Redox Regulation of Protein Functioning

**DOI:** 10.3390/biom13091281

**Published:** 2023-08-22

**Authors:** Andrey A. Zamyatnin

**Affiliations:** 1 Faculty of Bioengineering and Bioinformatics, Lomonosov Moscow State University, 119234 Moscow, Russia; zamyat@belozersky.msu.ru; 2 Belozersky Institute of Physico-Chemical Biology, Lomonosov Moscow State University, 119992 Moscow, Russia; 3 Research Center for Translational Medicine, Sirius University of Science and Technology, 354340 Sirius, Krasnodar Region, Russia; 4 Institute of Translational Medicine and Biotechnology, Sechenov First Moscow State Medical University (Sechenov University), 119991 Moscow, Russia

Reactive oxygen species (ROS) and their derivatives play a key role in signaling under normal and oxidative stress conditions in all aerobic living organisms. Most intracellular and intercellular signaling cascades interact with ROS-mediated pathways or are influenced by these highly reactive molecules. The molecular mechanisms of ROS signaling are usually based on protein sensing, resulting in modifications that affect their structure and function. This collection of original research articles and reviews presents a number of illustrative examples of the processes involved in the molecular sensing of ROS by proteins and the further transduction of ROS-initiated signals into cellular responses leading, in particular, to circadian clock functioning or stress responses of organisms to biotic and abiotic stimuli. The articles presented in this Special Issue also highlight particular examples of molecular cascades affected by ROS involved in human physiology and pathology and propose novel drug candidates.

Many plants represent economically important species used as sources of food, materials and particular compounds. Physiologically, plants can produce O_2_ themselves as a result of photosynthesis and therefore harbor some unique features that defend against the redundant activity of O_2_ and its derivatives—ROS. Thus, specific mechanisms of plants dealing with ROS support photosynthetic machinery and beyond, and, in particular, maintain response reactions to different biotic and abiotic stresses. Drought causes serious negative environmental impacts, causing yield losses of cultivated plants. In their study, Li et al. [1] identified a novel factor from banana plants that positively regulates plant drought stress resistance through modulating reactive oxygen species metabolism. Moreover, this study shows that this novel factor, which is represented by the autophagy-related ATG8 protein family member MaATG8f, enhances drought tolerance and links the regulation of oxygen species metabolism and abscisic acid biosynthesis to autophagy, supporting many eukaryotic adaptive reactions. Salt stress is another common abiotic stress affecting plant growth and development. Jedelská et al. [2] studied the role of S-nitrosoglutathione reductase in the interactions of key enzymes of ROS metabolism with reactive nitrogen species (RNS) mediated by protein S-nitrosation in responses to salinity and cadmium stress during tomato root development. In this study, the authors showed that root growth inhibition is a result of stress-triggered disruption of ROS homeostasis, which involves the modulation of RNS and S-nitrosation of NADPH oxidase and ascorbate peroxidase.

A review article by Aribisala and Sabiu [3] raises the challenges of developing a new generation of antimicrobials, many of which are represented by natural plant compounds that originally evolved as important components of plant responses to biotic stresses. A special class of antimicrobial drugs is represented by molecules that produce ROS to combat bacterial cells. Their mechanism of action is based on the generation of ROS, such as O_2_^•−^, ^•^OH and H_2_O_2_, leading to damaged cellular macromolecules—including proteins, nucleic acids and lipids—and thus the death of bacterial cells. By now, many such drugs have already found their applications, and descriptions of new ones are constantly being published. However, a number of issues related to the use of these drugs remain complex, in particular, safety issues, and unresolved.

ROS are key participants in signaling processes, underlying many physiological and pathological processes in the human body. Interestingly, one of the most prominent signs of aging is protein carbonylation mediated by oxidative stress [4]. One of the mechanisms of molecular circadian clock functioning is based on the half-life of circadian proteins. In their work, Baidanoff et al. [5] investigated the ability of circadian protein period 2 (PER2) to undergo oxidation of thiol groups of cysteine. In this work, the authors showed that cysteine oxidation of PER2 results in the formation of homodimers and multimers in HEK-293T cells. The authors suggest that this effect may be involved in the molecular tuning of the circadian clock. In a study by Vladimirov et al. [6], the redox-mediated formation of protein dimers is also involved in the normal functioning of photoreceptors. In this work, the authors showed that in a cellular model, oxidative stress induces the disulfide dimerization of recoverin as well as the phosphorylation of caveolin-1. Interestingly, the disulfide dimerization of recoverin was potentiated by zinc, which is a known mediator of redox homeostasis. Thus, the authors concluded that the formation of the signaling complex between recoverin and caveolin-1 is determined by redox regulation, whereas oxidative stress may disrupt the light-induced translocation of caveolin-1 into photoreceptors and affect rhodopsin desensitization.

Enzymes also represent targets for ROS and RNS, which often regulate their activity. In their review article [7], Petushkova and Zamyatnin consider the mechanisms underlying the regulation of redox-sensitive proteolytic enzymes, as well as their functions in maintaining cellular and extracellular homeostasis and, under certain conditions, triggering the processes of regulated cell death. The function of carbonic anhydrases containing Zn^2+^ atoms in their structure is to convert CO_2_ and H_2_O into bicarbonate and protons. Disorders associated with this enzyme lead to the development of glaucoma, as well as a number of cancers. In their work [8], Ahmed et al. describe the development of a number of inhibitors of the carbonic anhydrase as potential therapeutic agents. Based on results obtained from vitro and in in silico screening, the authors propose the most promising drug candidate.

The field of redox regulation of protein functioning is not limited to the topics covered in the articles published in this Special Issue; rather, it is a truly global field affecting all aspects of the life activity of aerobic living organisms. The research results in this field are not only of great fundamental importance, but also make it possible to create new technologies for medicine, veterinary, agriculture and in many other areas aimed at improving the quality of human life.
